# Cardamonin as a p38 MAPK Signaling Pathway Activator Inhibits Human Coronavirus OC43 Infection in Human Lung Cells

**DOI:** 10.3390/nu15061335

**Published:** 2023-03-09

**Authors:** Young-Hee Jin, Jung Sun Min, Sunoh Kwon

**Affiliations:** 1 KM Application Center, Korea Institute of Oriental Medicine, Daegu 41062, Republic of Korea; 2 KM Convergence Research Division, Korea Institute of Oriental Medicine, Daejeon 34054, Republic of Korea

**Keywords:** cardamonin, *Alpinia katsumadai*, human coronavirus, HCoV-OC43, p38 MAPK signaling

## Abstract

A natural chalcone, cardamonin (2′,4′-dihydroxy-6′-methoxychalcone; CDN) was isolated from the seeds of *Alpinia katsumadai* Hayata, which has been traditionally used to treat stomach aches. CDN has been reported to possess various pharmacological properties, including anticancer and anti-inflammatory effects. This study evaluated the antiviral activity of CDN against human coronavirus HCoV-OC43 and determined the mode of action in HCoV-OC43-infected human lung cell lines (MRC-5 and A549 cells). CDN significantly inhibited HCoV-OC43-induced cytopathic effects with an IC_50_ of 3.62 μM and a CC_50_ of >50 μM, resulting in a selectivity index of >13.81. CDN treatment reduced the level of viral RNA and the expression of spike and nucleocapsid proteins in HCoV-OC43-infected cells as determine through qRT-PCR and Western blot analysis. Additionally, the activation of p38 mitogen-activated protein kinase (MAPK) by anisomycin decreased viral protein expression, whereas an inhibitor of p38 MAPK signaling, SB202190, increased viral protein expression. CDN also amplified and extended the p38 MAPK signaling pathway in HCoV-OC43-infected cells. In conclusion, CDN inhibited HCoV-OC43 infection by activating the p38 MAPK signaling pathway and has potential as a therapeutic agent against human coronavirus.

## 1. Introduction

Coronaviruses (CoVs) are enveloped, positive-sense, and single-stranded RNA viruses belonging to the *Coronaviridae* family. They are divided into four genera: *Alphacoronavirus*, *Betacoronavirus*, *Gammacoronavirus*, and *Deltacoronavirus* [1]. Four human CoVs (HCoVs), HCoV-229E and HCoV-NL63 from *Alphacoronavirus* and HCoV-HKU1 and HCoV-OC43 from *Betacoronavirus* infect the human respiratory tract and cause 15%–29% of common colds [2]. In the last two decades, high-pathogenic zoonotic *Betacoronaviruses,* such as severe acute respiratory syndrome CoV (SARS-CoV), Middle East respiratory syndrome CoV, and SARS-CoV-2, have emerged [3,4] and been transmitted among humans, leading to acute respiratory distress syndrome and pandemics such as COVID-19 [5]. HCoV-OC43 of *Betacoronavirus*, discovered in 1967 and now globally distributed, is a well-studied prototype human coronavirus [6]. Similar to other viruses, HCoV hijacks various host cellular factors to modulate its life cycle. Researchers have used functional genetic and interactome screens to search cellular host factors that are required for infection as targets for broad-spectrum antiviral therapies [7]. In particular, HCoV is known to modulate cellular process such as apoptosis, the endoplasmic reticulum stress response, autophagy flux, innate immunity, and mitogen-activated protein kinase (MAPK) signaling [8,9,10].

The natural chalcone, cardamonin (2′,4′-dihydroxy-6′-methoxychalcone; CDN) is isolated from the seeds of *Alpinia katsumadai* Hayata, which belongs to the Zingiberaceae family and has traditionally been used as a remedy for stomach aches and diarrhea. CDN is known to possess various bioactive and pharmacological properties [11], such as anticancer activity through the regulation of Wint/β-catenin, PI3K/AKT, and NF-kB signaling [12], anti-inflammatory activity in LPS-stimulated monocytes/macrophages in vitro [13] and in a paw edema model in vivo [14], anticoagulative activity through the inhibition of platelet aggregation in human whole blood [15], antiviral activity through the inhibition of HIV-1 protease [16] and dengue virus type 2 NS3 protease [17] activity, antiparasitic activity against *Leishmania amazonensis* [18], and antifungal activity against *Epidermophyton floccosum* [19]. However, the effects of CDN on human CoVs remain unclear.

In this study, we evaluated the antiviral activity of CDN against human coronavirus HCoV-OC43 infection in the human lung cell lines MRC5 and A549. Subsequently, the CDN’s mechanism of action was further investigated. We found that CDN inhibits HCoV-OC43 infection by activating the p38 MAPK signaling pathway. These data suggest that CDN is potential therapeutic candidate for human coronavirus infection disease.

## 2. Materials and Methods

### 2.1. Compounds

Cardamonin (PubChem CID 641785) with ≧98% purity was purchased from Wuhan ChemFaces Biochemical Co., Ltd. (Wuhan, China). Anisomycin (PubChem CID 253602) and SB202190 (PubChem CID 5169) were purchased from Tocris Bioscience (Bristol, United Kingdom). These compounds were dissolved in dimethyl sulfoxide (DMSO, Sigma-Aldrich, St. Louis, MO, USA) at 20 mM and stored in a deep freezer at −80 °C.

### 2.2. Cells and Viral Infection

The human fetal lung fibroblast cell line, MRC-5, and human lung epithelial cell line, A549 [American Type Culture Collection (ATCC), Manassas, VA, USA], were cultured in Minimal Essential Medium (Corning Incorporated, Corning, NY, USA) with 10% fetal bovine serum (Gibco, Carlsbad, CA, USA) and 1% penicillin/streptomycin (Gibco) in an incubator at 37 °C with 5% CO_2_. HCoV-OC43 was obtained from ATCC and propagated and titrated as described previously [20]. MRC-5 and A549 cells were infected with 10^4.5^ TCID_50_/mL of HCoV-OC43 in an incubator at 33 °C with 5% CO_2_.

### 2.3. Assay of the CDN-Mediated Reduction in Virus-Induced Cytopathic Effects

MRC-5 cells (1 × 10^4^) were seeded in 96-well plates (Thermo Fisher Scientific Inc., Waltham, MA, USA) and incubated at 37 °C overnight. The cells were then infected with HCoV-OC43 and treated with serially diluted concentrations of CDN. At 4 days postinfection (dpi), viable cells were measured via an MTS assay (see Section 2.4.). HCoV-OC43-infected and vehicle-treated cells were used as positive and negative control cells, respectively. The final DMSO concentration did not exceed 0.1%.

### 2.4. MTS Assay

A colorimetric 3-(4,5-dimethylthiazol-2-yl)-5-(3-carboxymethoxyphenyl)-2-(4-sulfophenyl)-2H-tetrazolium (MTS) assay (Promega Corporation, Madison, WI, USA) was performed according to the manufacturer’s instructions. Absorbance was detected at 490 nm using a GloMax^®^ Discover Microplate Reader (GM3000, Promega).

### 2.5. HCoV-OC43 RNA Copy Number Quantification

Viral RNAs were collected from the supernatant and cell lysates of HCoV-OC43-infected MRC-5 cells using a QIAamp Viral RNA Mini Kit (Qiagen N.V., Hilden, Germany) and RNeasy^®^ Mini Kit (Qiagen), respectively, according to the manufacturer’s instructions. Isolated RNA was used to synthesize cDNA with a One Step TB Green^®^ PrimeScript™ RT-PCR Kit (Takara Bio Inc., Kusatsu, Japan) according to the manufacturer’s instructions. The copy number of HCoV-OC43 RNA was quantified using nucleocapsid protein RNA standards as described previously [20]. The primer sequences of HCoV-OC43 nucleocapsid proteins (N) used as standards are presented in Table 1.

### 2.6. Western Blot Analysis

Cells (1 × 10^5^ cells/24-well plate; Thermo Fisher Scientific Inc.) were infected with HCoV-OC43 and treated with the indicated compounds until 4 dpi at 33 °C. Cells lysates were then separated on SDS–PAGE gel and transferred to nitrocellulose filter membranes (Bio-Rad Laboratories, Inc., Hercules, CA, USA), which were incubated with 5% skim milk in TBST (0.5% Tween20) for 30 min at room temperature and then rinsed three times with cold TBST. The anti-HCoV-OC43 nucleocapsid protein antibody (Catalogue Number. MAB9012, Merck & Co., Inc., Rahway, NJ, USA), anti-HCoV-OC43 spike protein antibody (Cat. No. CSB-PA336163EA01HIY, Cusabio, Houston, TX, USA), anti-phospho-p38 MAPK antibody (Cat. No. 9211S, Cell Signaling Technology, Danvers, MA, USA), anti-total p38 MAPK antibody (Cat. No. 9212S, Cell Signaling Technology), or anti-β-actin antibody (Cat. No. 3700S, Cell Signaling Technology) was then added and incubated at 4 °C overnight. After washing, horseradish peroxidase-conjugated secondary antibodies (Abcam PLC, Cambridge, UK) were added and incubated for 2 h at room temperature. Proteins were detected using a ChemiDoc™ MP Imaging System (Bio-Rad Laboratories). Band intensity was measured using the ImageLab program (Bio-Rad).

### 2.7. Immunofluorescence Staining Analysis

Cells (1 × 10^5^ cells/24-well plate; Thermo Fisher Scientific Inc.) were grown on poly-L-lysine-coated coverslips overnight, fixed with 4% formaldehyde and permeabilized in PBS containing 0.2% Triton X-100. The cells were then blocked with 3% bovine serum albumin, and anti-HCoV-OC43 spike protein antibody (CusaBio) or anti-nucleocapsid antibody (Merck) was added and incubated at 4 °C overnight. Subsequently, the cells were rinsed with cold PBS and incubated with AlexaFluor555 goat-anti-rabbit IgG (Cat. No. A21428, Thermo Fisher Scientific Inc.) or AlexaFluor488 goat-anti-mouse IgG (Cat. No. A11001, Thermo Fisher Scientific Inc.) for 1 h. After washing with PBS, the cells were mounted with SlowFade Gold anti-fade reagent with DAPI (Invitrogen, Waltham, MA, USA) and visualized under a fluorescence microscopy (IX71, Olympus Corporation, Tokyo, Japan) with the cellSens Dimension program (Olympus).

### 2.8. Real-Time PCR for mRNA Quantitation

The total RNA of cells was isolated using a RNeasy^®^ Mini Kit (Qiagen) according to the manufacturer’s instructions. Extracted total RNA was used to synthesize cDNA, which was amplified using a One Step TB Green^®^ PrimeScript^TM^ RT-PCR Kit (Takara Bio) according to the manufacturer’s instructions. The primers used in these experiments are listed in Table 1.

### 2.9. Cytometric Bead Array Analysis

Six inflammatory cytokines (interleukin (IL)-1β, IL-6, IL-8, IL-10, IL-12p70, and tumor necrosis factor) were detected in culture supernatants using a BD™ CBA Human Inflammatory Cytokines Kit and BD LSRFortessa X-20 Flow Cytometer (BD Biosciences, San Jose, CA, USA). The resultant data were analyzed using FCAP Array™ Software version 3.0 (Soft Flow Hungary, Ltd., Pécs, Hungary) and the standard curves of recombinant cytokine standards.

### 2.10. Statistical Analysis

Statistical analyses were performed using GraphPad Prism version 9.5.0 (GraphPad Software Inc., San Diego, CA, USA). All data are presented as the mean ± standard error of the mean. One- or two-way ANOVA followed by Bonferroni’s multiple comparison was used to determine statistical differences. *p* values of <0.05 were considered statistically significant.

## 3. Results

### 3.1. CDN Protected Cells from HCoV-OC43-Induced Cytopathic Effects

**Figure 1:**
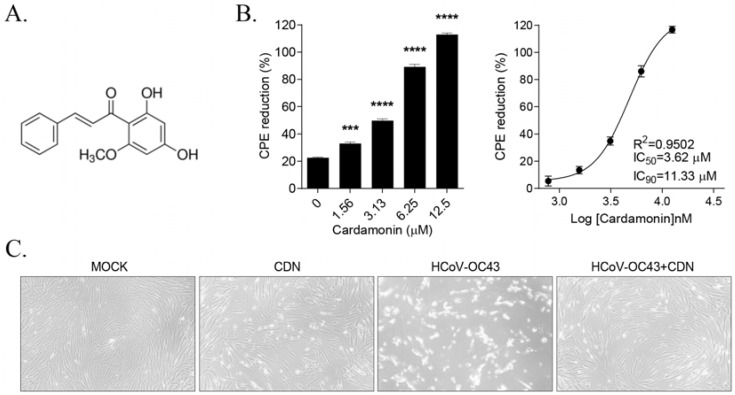
Cardamonin (CDN) inhibited HCoV-OC43-induced cytopathic effects (CPEs). (**A**) Chemical structure of CDN. (**B**) CPE of CDN against HCoV-OC43 infection. MRC-5 human lung fibroblast cells were infected with 10^4.5^ TCID_50_/mL HCoV-OC43 and treated with two-fold serially diluted concentrations of CDN. At 4 days postinfection (dpi), the reduction in CPEs was measured using an MTS assay (**left** panel). *p* values were determined via one-way ANOVA followed by Bonferroni’s multiple comparisons test (*** *p* < 0.001 and **** *p* < 0.0001 vs. 0 μM CDN). The IC_50_ and IC_90_ values of CDN were 3.62 and 11.33 μM, respectively, as determined by nonlinear regression analysis (**right** panel). Data represent the means ± standard error of three independent experiments. (**C**) Images of mock, 10 μM CDN-treated, HCoV-OC43-infected, and virus-infected and 10 μM CDN-treated cells were captured at 4 dpi.

MRC-5 cells were infected with HCoV-OC43 and treated with serially diluted concentrations of CDN (Figure 1A) from 1.56 to 12.50 μM. At 4 dpi, HCoV-OC43 infection induced cytopathic effects (CPEs) up to ~80% cell death, as determined via an MTS assay. However, CDN treatment dose-dependently inhibited virus-induced CPEs in cells, and CDN at 12.50 μM elicited 100% inhibition of virus-induced CPEs. The 50% and 90% maximal inhibitory concentrations (IC_50_ and IC_90_, respectively) were 3.62 and 11.33 μM, respectively, and the 50% cytotoxicity concentration (CC_50_) was >50 μM, resulting in a selectivity index (CC_50_/IC_50_) of >13.81 (Figure 1B). The inhibition of virus-induced CPEs in 10 μM CDN-treated cells is shown in Figure 1C. Overall, these results indicate that CDN treatment significantly inhibits virus-induced CPEs, suggesting that CDN has the potential to exert anti-HCoV-OC43 effects.

### 3.2. CDN Inhibited HCoV-OC43 Replication and Viral Protein Expression in Human Lung Cells

**Figure 2:**
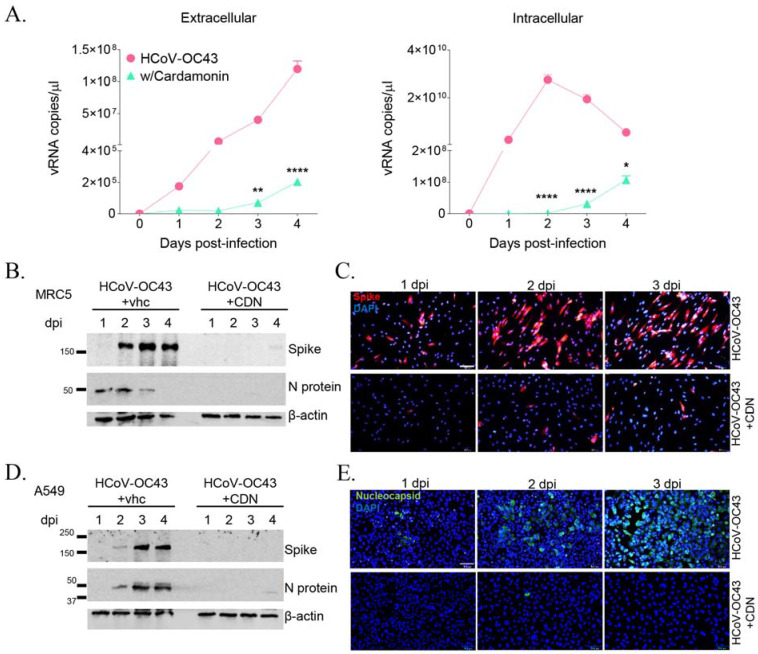
Cardamonin (CDN) inhibited HCoV-OC43 replication and viral protein expression. (**A**) Viral RNA levels in the culture supernatant (Extracellular, **left** panel) and cell lysate (Intracellular, **right** panel) of virus-infected MRC-5 cells with (triangle) and without (circle) 10 μM CDN treatment were assessed at 1–4 dpi using qRT-PCR. Two-way ANOVA followed by Bonferroni’s multiple comparisons test was used to compare the resultant data (* *p* < 0.05, ** *p* < 0.01, and **** *p* < 0.0001 vs. HCoV-OC43), which are presented as the mean ± standard error of the mean. (**B**,**D**) The expression of viral proteins, i.e., spike and nucleocapsid (N) proteins, in MRC-5 (**B**) and A549 (**D**) cells was detected using Western blot analysis following HCoV-OC43 infection and vehicle or 10 μM CDN treatment at 1–4 dpi. β-actin was used as the internal control. (**C**,**E**) Immunofluorescence staining images captured at 1–3 dpi. The spike protein (red) of HCoV-OC43 and nucleus with DAPI (blue) in MRC-5 cells (**C**) and nucleocapsid protein (green) and nucleus with DAPI (blue) in A549 cells (**E**) were detected following viral infection and treatment with vehicle or 10 μM CDN. Scale bar: 100 μm. At least three independent experiments were performed in each case.

The anti-HCoV-OC43 activity of CDN was evaluated via qRT-PCR by assessing the viral RNA copy number following treatment with 10 μM CDN in HCoV-OC43-infected MRC-5 cells. The viral RNA copy number in virus-infected culture supernatants increased during the 4-day infection period to up to 1 × 10^8^/μL copies. However, treatment with 10 μM CDN significantly decreased the viral RNA copy number to around 2 × 10^5^/μL copies (Figure 2A, left panel). The amount of viral RNA in virus-infected cell lysate peaked at 2 dpi at >2 × 10^10^/μL copies, whereas CDN-treated cell lysates exhibited a low viral copy number of 1 × 10^6^/μL copies at 2 dpi (Figure 2A, right panel). These results indicate that CDN significantly decreases the viral RNA level in both the culture supernatant and cell lysate of MRC-5 cells.

To further explore the inhibitory effect of CDN on HCoV-OC43 infection, the expression of viral proteins was assessed after CDN treatment in virus-infected MRC-5 and A549 cells. According to the Western blot analysis, the spike and nucleocapsid proteins were detected in virus-infected cells, but these proteins were virtually undetected in 10 μM CDN-treated HCoV-OC43-infected MRC5 (Figure 2B) and A549 (Figure 2D) cells. The expression levels of spike protein in MRC5 cells (Figure 2C) and nucleocapsid protein in A549 cells (Figure 2E) treated with vehicle or CDN were also evaluated at 1–3 dpi using an immunofluorescence staining assay. The results were consistent with those of the Western blot analysis, i.e., 10 μM CDN inhibited viral protein expression levels. These findings indicate that CDN inhibits the replication of HCoV-OC43 and the expression of the viral proteins in the human lung cell lines MRC-5 and A549.

### 3.3. CDN Did Not Induce a Host IFN-Related Antiviral Response during HCoV-OC43 Infection

**Figure 3:**
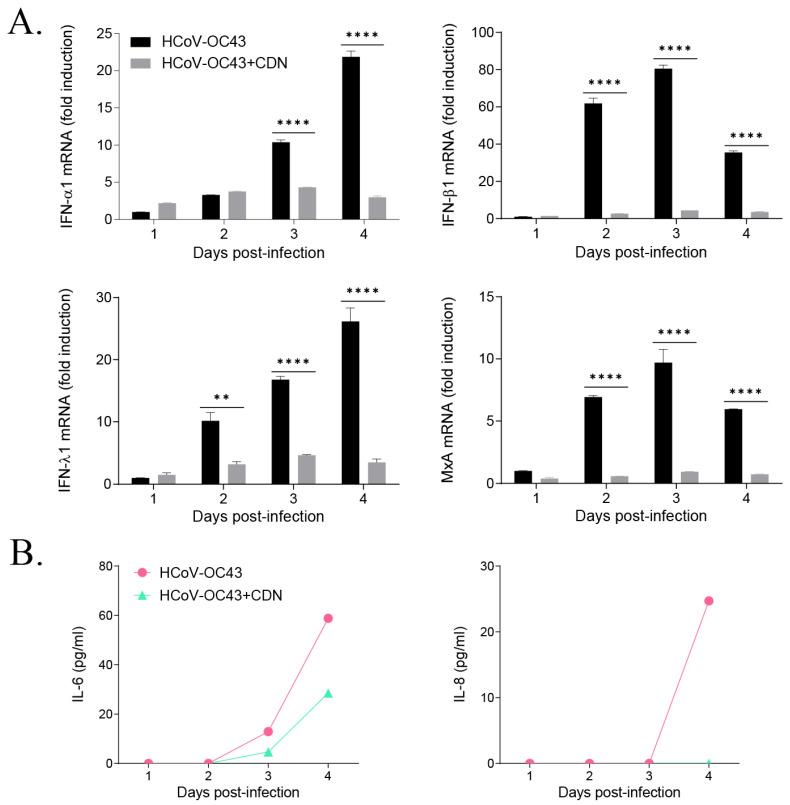
Cardamonin (CDN) treatment did not enhance the host IFN-related antiviral response during HCoV-OC43 infection. (**A**) The gene expression levels of *IFN-α*, *IFN-β*, *IFN-λ*, and *MxA* were quantified in HCoV-OC43-infected MRC-5 cells treated with vehicle (black bar) or 10 μM CDN (gray bar) at 1–4 dpi using qRT-PCR. Data were normalized relative to the *β-actin* gene expression level. Two-way ANOVA followed by Bonferroni’s multiple comparisons test was used to compare the data (** *p* < 0.01 and **** *p* < 0.0001 vs. HCoV-OC43). (**B**) Levels of the proinflammatory cytokines, IL-6, and IL-8, secreted in virus-infected MRC-5 cell culture supernatant treated with vehicle (circle) or 10 μM CDN (triangle), were determined using a cytometric bead array kit. Data represent the means ± standard error of the mean of two independent experiments.

To investigate the mechanism underlying the antiviral effects of CDN on HCoV-OC43 infection, the expression levels of IFN-related antiviral genes, *IFN-α*, *IFN-β*, *IFN-λ*, and *MxA*, were examined in CDN-treated MRC-5 cells via qRT-PCR. The expression levels of these genes increased following HCoV-OC43 infection at 1–4 dpi. However, CDN-treated cells did not exhibit an IFN-related gene expression response, possibly owing to the low virus load in these cells (Figure 3A). Secretion of the proinflammatory cytokines IL-6 and IL-8 was increased in HCoV-OC43-infected MRC-5 cells; however, the levels of these cytokines were reduced in CDN-treated cells, consistent with the IFN-related gene response (Figure 3B). These findings suggest that CDN does not induce IFN-related gene expression and proinflammatory cytokine secretion during HCoV-OC43 infection.

### 3.4. CDN Amplified and Extended HCoV-OC43-Induced p38 Phosphorylation

**Figure 4:**
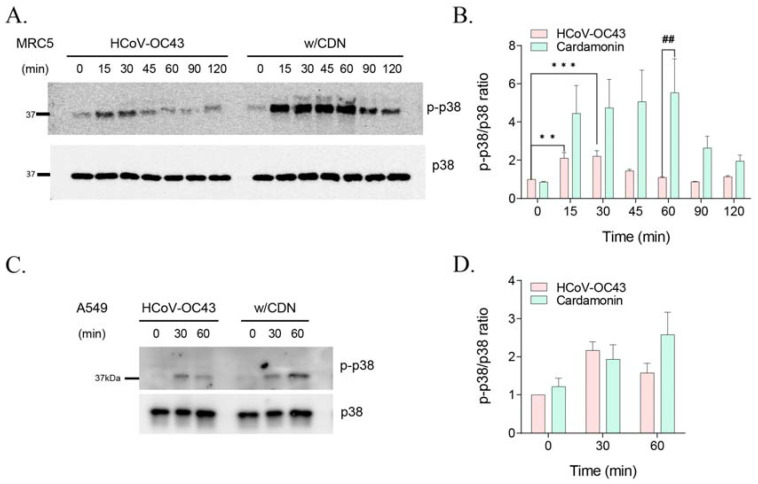
Cardamonin (CDN) activated the p38 MAPK signaling pathway in HCoV-OC43-infected human lung cell lines. (A and C) MRC-5 (**A**) and A549 (**C**) cells were infected with HCoV-OC43 and treated with vehicle or 10 μM CDN for the indicated time. Phosphorylation of p38 MAPK (p-p38) and total p38 MAPK (p38) were then detected using a Western blot assay. (**B**,**D**) The ratio of p-p38 levels to total p38 was measured using the Western blot data in (**A**,**C**), respectively, via ImageLab. Two-way ANOVA followed by Bonferroni’s multiple comparisons test was used analyze the data (CDN effect, *p* < 0.0001; time effect, *p* = 0.0255; CDN × time interaction, not significant) (** *p* < 0.01 and *** *p* < 0.001 vs. HCoV-OC43 at 0 min; ^##^ *p* < 0.01 vs. HCoV-OC43 at 60 min). Data represent at least two independent experiments.

CDN has been found to activate the p38 MAPK signaling pathway [21,22], and HCoV-OC43 infection in MRC-5 cells was previously found to mediate the phosphorylation of p38 MAPK [20]. Therefore, we evaluated the CDN-mediated expression level of phosphorylated p38 MAPK in MRC5 and A549 cells during HCoV-OC43 infection. Western blot analysis revealed that HCoV-OC43 infection in MRC-5 cells induced the phosphorylation of p38 MAPK as early as 15 min postinfection and maintained at 30 min postinfection, and phosphorylation levels decreased from 45 min postinfection. Treatment with 10 μM CDN increased the phosphorylation of p38 MAPK by approximately two-fold relative to that in virus-infected cells at 15 min postinfection, and the increased phosphorylation levels were maintained until 60 min postinfection (Figure 4A,B). Similarly, in A549 cells, CDN treatment increased the phosphorylation of p38 MAPK by approximately 1.6-fold relative to that in virus-infected cells until 60 min postinfection, whereas virus-infected cells exhibited peak phosphorylation until 30 min postinfection (Figure 4C,D). These findings suggest that CDN amplifies and extends HCoV-OC43-induced activation of the p38 MAPK signaling pathway in human lung cell lines.

### 3.5. p38 MAPK Activation by CDN and/or Anisomycin Suppressed HCoV-OC43 Infection

**Figure 5:**
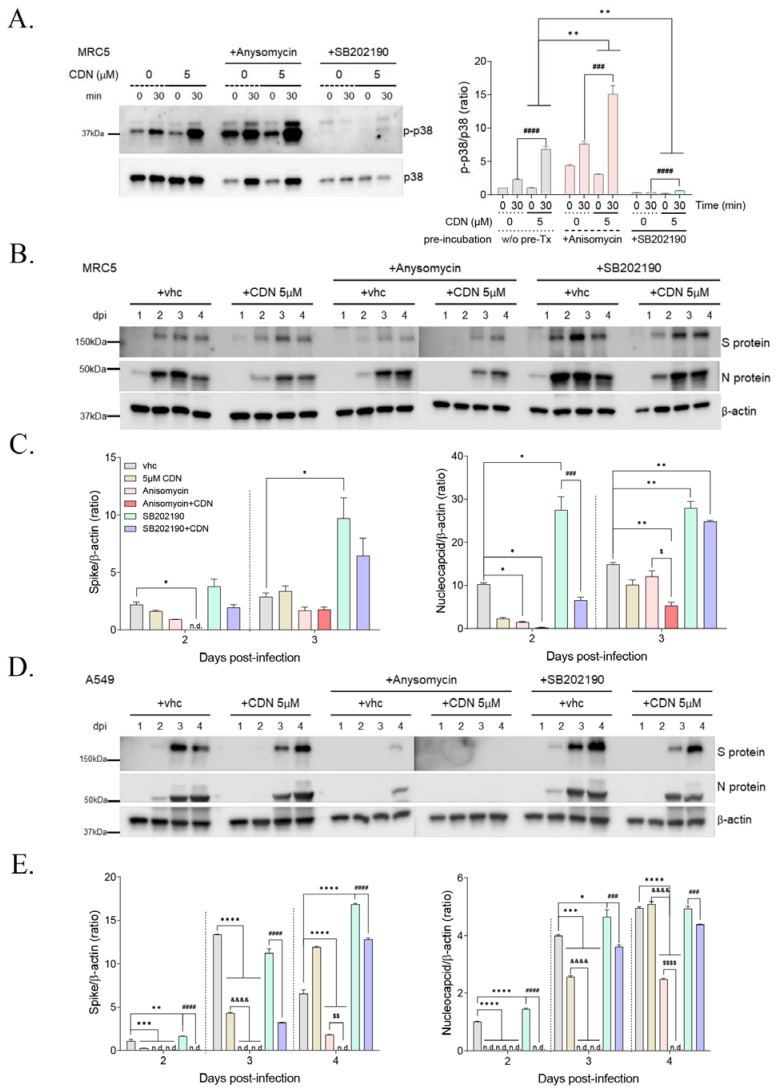
Activation of the p38 MAPK signaling pathway by CDN suppressed HCoV-OC43 infection. (**A**) MRC-5 cells were preincubated with 0.1 μM anisomycin or 0.1 μM SB202190 for 1 h and then infected with HCoV-OC43 with or without a suboptimal concentration of cardamonin (CDN; 5 μM). The phosphorylation of p38 MAPK (p-p38) and total p38 MAPK (p38) was detected at 30 min postinfection using a Western blot assay (left panel). The ratio of p-p38 levels to total p38 was measured using ImageLab (right panel). Two-way ANOVA followed by Bonferroni’s multiple comparison was used to compare data (** *p* < 0.01 vs. 5 μM CDN w/o pre-Tx; ^###^ *p* < 0.001 and ^####^ *p* < 0.0001 vs. 0 μM CDN). (**B**–**E**) Under the same conditions, viral spike (S) and nucleocapsid (N) proteins were detected at 1–4 dpi in MRC-5 cells (**B**) and A549 cells (**D**) using a Western blot assay. β-actin was used as the internal control. (**C**,**E**) Ratios of viral spike (**left** graph) and nucleocapsid (**right** graph) levels relative to control levels (β-actin) in MRC-5 cells (**C**) and A549 cells (**E**) were measured using ImageLab. One-way ANOVA followed by Bonferroni’s multiple comparison was used to compare data (* *p* < 0.05, ** *p* < 0.01, *** *p* < 0.001, and **** *p* < 0.0001 vs. vhc; ^&&&&^ *p* < 0.0001 vs. 5 μM CDN; ^$^ *p* < 0.05, ^$$^ *p* < 0.01, and ^$$$$^ *p* < 0.0001 vs. anisomycin; ^###^ *p* < 0.001 and ^####^ *p* < 0.0001 vs. SB202190). Data represent the mean ± standard error of the mean of at least three independent experiments. n.d., not detected.

To further investigate the role of CDN-mediated p38 MAPK activation during HCoV-OC43 infection, p38 MAPK activation was amplified or suppressed using the p38 MAPK activator anisomycin or p38 MAPK inhibitor SB202190, respectively, in HCoV-OC43-infected MRC-5 cells with or without a suboptimal CDN treatment (5 μM). MRC-5 cells were preincubated with 0.1 μM anisomycin or 0.1 μM SB202190 for 1 h and then infected with HCoV-OC43 with or without 5 μM CDN. The phosphorylation of p38 MAPK was then detected using Western blot analysis to confirm the activation or suppression of p38 MAPK at 30 min postinfection (Figure 5A). Viral infection was found to induce a 2-fold induction in the phosphorylation of p38 MAPK, whereas viral infection with CDN treatment led to a 6-fold induction, viral infection with anisomycin treatment led to a 7-fold induction, and viral infection with anisomycin and CDN treatments led to a 14-fold induction. In contrast, SB202190 treatment led to a 0.2-fold induction, viral infection with SB202190 treatment led to a 0.3-fold induction, and viral infection with SB202190 and CDN treatments led to a 0.6-fold induction in the phosphorylation of p38 MAPK. These findings indicated that anisomycin and SB202190 were effective p38 MAPK activators and inhibitors, respectively, and that viral infection with CDN treatment significantly induced the activation of p38 MAPK under both anisomycin- and SB202190-treated conditions.

Under the same conditions, we used Western blot analysis to evaluate the expression level of viral spike and nucleocapsid proteins and determine the viral load at 1–4 dpi in MRC-5 and A549 cells (Figure 5B–E). We found that even a suboptimal concentration of CDN (5 μM) slightly reduced the expression level of viral spike and nucleocapsid proteins in both cell lines. Given that anisomycin treatment increased the phosphorylation of p38 MAPK, viral protein expression levels were markedly reduced in both MRC-5 and A549 cells, suggesting that p38 MAPK activation suppressed viral protein expression. A treatment of anisomycin with CDN also synergically increased the phosphorylation of p38 MAPK, and viral protein expression levels were almost diminished at 2 dpi in MRC-5 cells and at 1–4 dpi in A549 cells. In contrast, treatment with the p38 MAPK inhibitor SB202190 abolished the phosphorylation of p38 MAPK and increased the expression levels of viral proteins. During the SB202190 treatment, CDN treatment still increased the phosphorylation of p38 MAPK and reduced viral protein expression more than with only SB202190 treatment. Therefore, activation of the p38 MAPK signaling pathway inhibits HCoV-OC43 infection. CDN can inhibit HCoV-OC43 infection through activation of the p38 MAPK signaling pathway.

## 4. Discussion

As a natural chalcone, CDN is known to possess a range of pharmacological activities, including anti-inflammation and anticancer effects, as well as strong biosafety and pharmacokinetic profiling; therefore, it is considered a promising compound for drug development [12]. In the present study, we examined the antiviral activity of CDN against human coronavirus HCoV-OC43, finding that its IC_50_ value and selectivity index were 3.62 μM and >13.81, respectively, owing to the compound activating the p38 MAPK signaling pathway.

Although cellular antiviral factors can limit viral infection, viruses are known to exploit cellular host factors and signaling pathways for their life cycle, e.g., during entry, translation, replication, transcription, and virion packaging [23]. Viruses are known to modulate cellular processes, including the MAPK family, which consists of extracellular signal-regulated kinases, Jun N-terminal kinases, and p38 MAPK [24]. Many viruses have been reported to activate the p38 MAPK signaling pathway, including hepatitis C, severe fever with thrombocytopenia syndrome, herpes simplex, SARS-CoV-2 [25], influenza A [26], respiratory syncytial [27], hepatitis B [28], and Ebola [29] viruses. It was shown that such viral infections were inhibited by p38 MAPK inhibitors; therefore, these inhibitors are considered potential drugs for treating viral infection-related diseases [30].

We also found that HCoV-OC43 infection induced the activation of p38 MAPK signaling at early time of infection in human lung cell lines (Figure 4). However, when a 1 h pretreatment of the p38 MAPK inhibitor SB202190 was used to abrogate the phosphorylation of p38 MAPK, viral protein expression levels were increased significantly in human lung cell lines (Figure 5). Moreover, the p38 MAPK signaling pathway was amplified via a 1 h anisomycin treatment preincubation, and viral protein expression levels were decreased significantly. Based on these findings, we conclude that activation of p38 MAPK signaling can mediate the inhibition of HCoV-OC43 infection under the experimental conditions of our study.

CDN is known to exert the anticancer activity via activation of the p38 MAPK signaling pathway in cancer cell lines [21,22]. Our findings indicate that CDN also exerts antiviral bioactivity against HCoV-OC43 infection (Figure 1 and Figure 2). These antiviral effects were induced by CDN through amplification and extension of the p38 MAPK signaling pathway in HCoV-OC3-infected human lung cell lines (Figure 4). Given the validatory findings with CDN following pretreatments with the p38 MAPK activator and inhibitor, anisomycin and SB202190, respectively (Figure 5), our results strongly suggest that the anti-HCoV-OC43 infection activity of CDN is exerted through p38 MAPK signaling pathway activation, which likely plays a role as a cellular antiviral factor.

In this study, we conclude that CDN activates the activation of the p38 MAPK signaling pathway, which inhibited HCoV-OC43 infection under the experimental conditions of our study. Therefore, we suggest that CDN has potential as an antiviral therapeutic agent and warrants further investigation in relation to drug development using structure activity relationship studies based on the antiviral activity of CDN.

## Figures and Tables

**Table 1:** Primer sequences for qRT-PCR.

Gene	Forward	Reverse
N	AGCAACCAGGCTGATGTCAATACC	AGCAGACCTTCCTGAGCCTTCAAT
IFN-α1	GTGCTCAGCTGCAAGTCAAG	TTATCCAGGCTGTGGGTCTC
IFN-β1	ACCAACAAGTGTCTCCTCCA	GTAGTGGAGAAGCACAACAGG
IFN-λ1	GTCACCTTCAACCTCTTCCG	TCAGACACAGGTTCCCATCG
MxA	CAACCTGTGCAGCCAGTATG	GTCCTGCTCCACACCTAGAG
β-actin	GGAAATCGTGCGTGACATCA	ATCTCCTGCTCGAAGTCCAG

## Data Availability

Not applicable.
